# Role of Contrast-Enhanced Ultrasound in the Evaluation of Patients With Suspected Renal Arterial Stenosis

**DOI:** 10.3389/fcvm.2022.721201

**Published:** 2022-03-04

**Authors:** Yang Wang, Yan Li, Siyu Wang, Na Ma, Junhong Ren

**Affiliations:** Department of Ultrasound, Beijing Hospital, National Center of Gerontology, Institute of Geriatric Medicine, Chinese Academy of Medical Sciences, Beijing, China

**Keywords:** renal artery stenosis (RAS), CEUS (contrast-enhanced ultrasound), ultrasonography, Doppler ultrasound, angiography

## Abstract

**Objectives:**

To explore the role of Contrast-enhanced ultrasound (CEUS) in the evaluation of patients with suspected renal artery stenosis and analyze the causes of the misdiagnosis and missed diagnosis.

**Methods:**

The data of 40 patients (80 renal arteries) diagnosed with RAS by CEUS in Beijing Hospital from September 2018 to October 2020 were compared with their digital subtraction angiography (DSA) results to analyze the causes underlying missed diagnosis and misdiagnosis of RAS by CEUS.

**Results:**

1. Compared with the gold standard DSA results, the AUC of the ROC curve of CEUS in detecting normal renal artery and renal artery stenosis was 0.961, the sensitivity was 96.4%, the specificity was 95.8%, and the Kappa value of the consistency analysis was 0.912 (*P* < 0.01); 2. Compared with the gold standard DSA results, the ROC curve of CEUS in distinguishing renal artery stenosis ≥70% from <70% stenosis has an AUC of 0.916, a sensitivity of 90.9%, a specificity of 92.3%, and the Kappa value of the consistency analysis is 0.77 (*P* < 0.01); 3. CEUS missed two cases (one for mild stenosis and one for moderate stenosis), and the detection rate of renal artery stenosis was 97.5% (78/80); A total of 65 renal arteries diagnosed by CEUS were consistent with DSA, and the diagnostic accuracy of CEUS for the degree of stenosis was 81.25% (65/80); Among the 13 misdiagnosed renal arteries, 4 of them can be corrected to the same degree as DSA by the reference to hemodynamic index, and the diagnosis rate of the degree of renal artery stenosis by ultrasonography (combined with CEUS and hemodynamic indicators) can be improved to 86.25%.

**Conclusions:**

1. CEUS can clearly show the renal arteries, and is consistent with DSA in distinguishing normal renal artery stenosis from renal artery stenosis, as well as renal artery stenosis ≥70% and <70% stenosis; 2. CEUS showed good performance in detecting normal renal artery and renal artery stenosis, and the missed diagnosis is concentrated on mild and moderate stenosis; 3. CEUS combined with hemodynamic indicators (Doppler ultrasound) can improve the accurate diagnosis rate of renal artery stenosis by ultrasonography; 4. The most important factor for the accurate diagnosis of renal artery stenosis by CEUS is the operator's standardized examination, which is not only related to the duration of the operator has been engaged in this inspection, but also related to whether the operator has received professional training in relevant aspects. These all indicate the necessity and importance of the standardized operation of renal artery contrast-enhanced ultrasound examination, and professional training should be given to operators.

## Introduction

Renal artery stenosis (RAS), which may lead to refractory hypertension and/or renal insufficiency, has been a substantial clinical concern (1). Digital subtraction angiography (DSA) is the gold standard for RAS diagnosis (2), but it is not a preferred imaging examination method due to its invasive nature and radiation exposure (3). In addition, iodinated contrast agents (ICAs) used in computed tomography angiography (CTA) and magnetic resonance angiography (MRA) carry the risk of contrast-induced nephropathy (4, 5). Instead, contrast-enhanced ultrasound (CEUS), which uses contrast agents without hepatorenal toxic side effects, is especially suitable for patients allergic to ICAs or with renal insufficiency (6). Ultrasound, the first-line imaging examination for RAS screening, commonly uses hemodynamic parameters for diagnosis (7), including peak systolic velocity (PSV), abdominal aortic velocity, the renal-aortic ratio (RAR) of the PSV and the presence of tardus-parvus renal artery spectral waveforms. However, these parameters are affected by various factors, including metabolic status, blood pressure, vascular wall compliance, traveling course of the renal arteries and renal parenchymal lesions. In addition, the diagnostic thresholds of hemodynamic parameters for RAS are inconsistent among existing studies. The present study primarily used CEUS for RAS diagnosis; this approach determines the degree of RAS based on the morphology of the renal arteries, i.e., the change in vascular luminal diameter, and thus, the CEUS-based diagnostic approach is based on principles and processes which is similar to DSA for the diagnosis of RAS.

## Materials and Methods

### Subjects

This study included a cohort of 40 patients, including 24 males and 16 females, who were diagnosed with RAS by CEUS in Beijing Hospital between September 2018 and October 2020. The ages of the patients ranged from 18 to 84 years old (average age 60.98 ± 17.81 years old), and the body mass index (BMI) of the patients ranged from 17.30 to 30.86 kg/m^2^ (average BMI 24.20 ± 3.61 kg/m^2^). All patients received DSA following CEUS. The inclusion criteria were: (1). patients who were diagnosed with RAS on either one or both sides by CEUS and re-examined later by DSA; and (2). patients who did not undergo CTA, MRA, or DSA prior to CEUS. After excluding patients with incomplete clinical data, 40 patients (80 renal arteries) were included in the final analysis in the study.

### Instruments and Methods

#### Instruments

The instrument used for ultrasonic diagnosis was a Samsung RS80A ultrasound scanner with a CA1-7 convex array transducer (Samsung Medison Co. Ltd., Seoul, Korea); the transducer frequencies ranged from 2 to 5 MHz. SonoVue (Bracco Company, Italy) was used as the contrast agent. The settings for renal artery CEUS were as follows: mechanical index (MI), 0.079; gain, 60 dB; and sensitivity, PEN2.

#### Methods

The patients, who had fasted for more than 8 hours, were placed in the supine, left, and right decubitus positions for routine ultrasound scanning and CEUS of the renal arteries. Routine ultrasound scanning covered the abdominal aorta (near the beginning of the renal artery), the main renal artery trunk (from its beginning to the renal hilum) and the internal renal artery (segmental and interlobular arteries). First, grayscale and color Doppler ultrasound were performed to measure the number of renal artery branches originating from the abdominal aorta, the luminal diameter of the renal arteries, the presence of plaques in the renal arterial wall, the renal artery blood flow status, as well as the PSV, resistive index and acceleration time in the renal artery at different locations. Subsequently, CEUS was used to display the entire main renal artery trunk. After the transducer was fixed into position, ensuring that the main renal artery trunk was displayed as much as possible while clearly demonstrating the starting point of the renal artery, the contrast-enhanced imaging process was initiated. Following the bolus injection of 1 ml of SonoVue into the cubital vein, 5 ml of 0.9% sodium chloride was injected to quickly flush the cannula. Recording was started at the same time of contrast agent injection, and 30 seconds of dynamic imaging was recorded. The CEUS results were compared with the DSA results for the presence and degree of stenosis in all 80 renal arteries.

For all patients, CEUS assessments were completed by a team comprising a senior physician and a junior physician from our ultrasound angiography department.

The diagnostic criteria of RAS included both hemodynamic and morphological diagnostic criteria. This study used morphological parameters obtained from CEUS as the diagnostic criterion, i.e., the degree of stenosis = [(1 – the diameter of the residual stenotic lumen)/normal luminal diameter] × 100%. A degree of stenosis <30% was classified as normal, ≥30% but <50% was classified as mild, ≥50% but <70% was classified as moderate, and ≥70% but ≤ 99% was classified as severe; no blood flow indicated complete occlusion of the renal artery. The hemodynamic parameters obtained from Doppler ultrasound (DUS) included PSV in the stenotic lumen, PSV in the abdominal aorta, RAR, and the presence of tardus-parvus renal artery spectral waveforms.

### Statistical Methods

SPSS 23.0 statistical software was used for data analysis. Measurement data with a normal distribution are expressed as the mean ± standard deviation. Count data are expressed as the number of cases (percentage) and were compared between groups using the chi-squared test. Kappa tests were conducted for consistency analyses; a Kappa value ≥0.75 indicated that the consistency was satisfactory, and a Kappa value ≥0.4 but <0.75 indicated that the consistency was barely acceptable. A *P* value < 0.05 indicated a statistically significant difference.

## Results

All 40 patients (80 renal arteries) were diagnosed with RAS by CEUS and subsequently underwent DSA. Using the DSA results as standards for comparison, CEUS missed two cases of RAS, for a detection rate of 97.5% (78/80). CEUS and DSA findings were consistent in determining the degree of stenosis in 65 renal arteries, showing an accuracy rate of 81.25% (65/80). The missed diagnosis or misdiagnosis of 15 renal arteries by CEUS could be attributed to one of the following three reasons. First, for the cases in which hyperechoic plaques were presented in the arterial wall at stenotic sites, the blockage of the plaques resulted in relatively narrow contrast agent echoes in the residual renal lumen, leading to an overestimation of stenosis (3/15). Second, CEUS generates two-dimensional images, and therefore, plaques in the lateral wall could be easily missed (2/15). Third, in patients with remodeled renal arteries, the normal luminal diameter could be inaccurately measured (usually underestimated), and as a result, the degree of stenosis could have been overestimated by CEUS (2/15). Various ultrasound parameters can be used for the diagnosis of RAS. While DUS is most commonly used, CEUS provides a morphological assessment (8, 9). The diagnostic parameters mainly include PSV, RAR, and the presence of tardus-parvus renal artery spectral waveforms. Among the 13 renal arteries misdiagnosed by CEUS, the degree of stenosis could be corrected to be consistent with the DSA results in four renal arteries when DUS parameters were considered. In other words, the diagnostic approach combining CEUS and DUS achieved an accuracy rate of 86.25% (69/80) for RAS. Because the renal arteries are located deep in the abdomen and have a small diameter, the quality of ultrasound images is easily affected by patient body shape and intestinal gas in the abdominal area, requiring the operator to be highly skilled. To achieve the highest quality ultrasound images of the renal arteries, all CEUS examinations in this study were conducted by a team comprising a senior physician and a junior physician from the angiography group in the ultrasound department, of which all members have received professional training for standardized renal artery examination. The examination process and key elements of renal artery CEUS were consistent.

**Figure 1:**
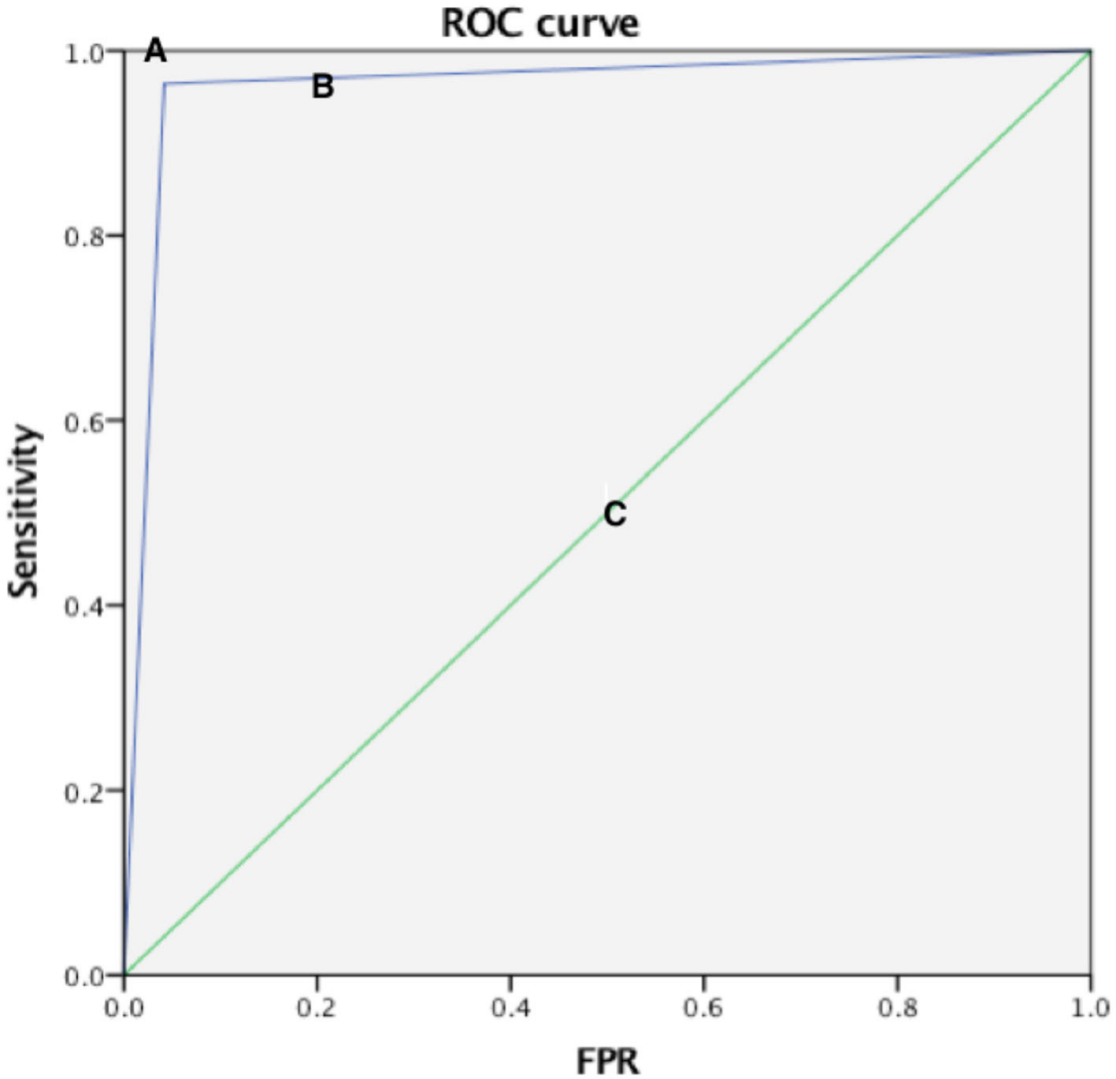
Three ROC curves with different values of the area under the ROC curve. A perfect test **(A)** has an area under the ROC curve of 1. The chance diagonal [**(C)**, the line segment from 0, 0 to 1, 1] has an area under the ROC curve of 0.5. The ROC curve tested can distinguish subjects with normal and narrow renal arteries **(B)**. The large area under ROC curve shows that test B has good diagnostic performance.

**Figure 2:**
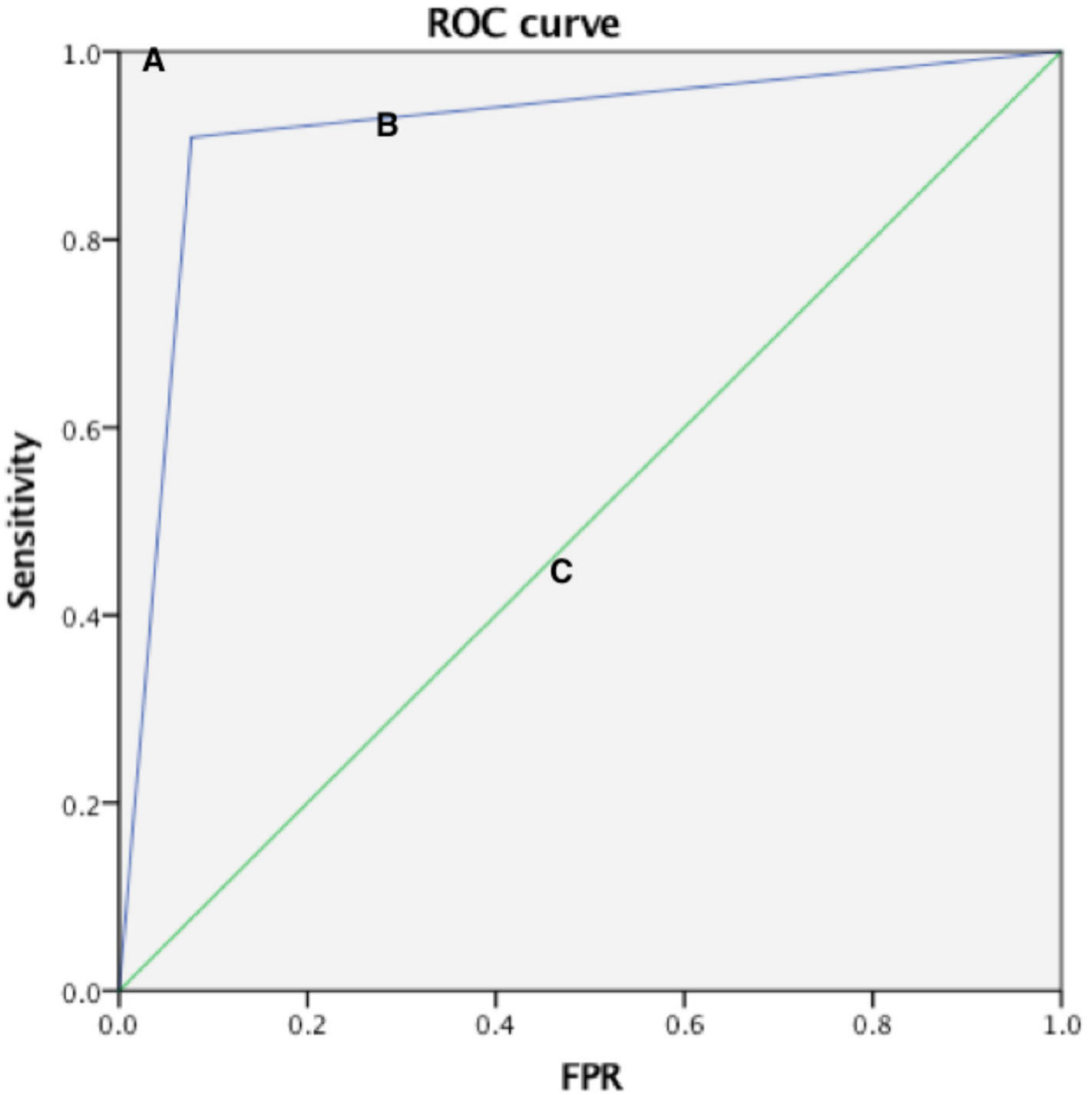
Three ROC curves with different values of the area under the ROC curve. A perfect test **(A)** has an area under the ROC curve of 1. The chance diagonal [**(C)**, the line segment from 0, 0 to 1, 1] has an area under the ROC curve of 0.5. The ROC curve tested can distinguish subjects with renal artery stenosis ≥70% and <70% **(B)**. The large area under ROC curve shows that test B has good diagnostic performance.

Compared with the gold standard DSA results, the AUC of the ROC curve of CEUS in detecting normal renal artery and renal artery stenosis was 0.961, the sensitivity was 96.4%, the specificity was 95.8%, and the Kappa value of the consistency analysis was 0.912 (*P* < 0.01) (Figure 1); in addition, the ROC curve of CEUS in distinguishing renal artery stenosis ≥70% from <70% stenosis has an AUC of 0.916, a sensitivity of 90.9%, a specificity of 92.3%, and the Kappa value of the consistency analysis is 0.77 (*P* < 0.01), which shows that CEUS diagnosis is very effective (Figure 2). This suggests that CEUS plays an important role in the detection of renal artery stenosis when investigating the cause of hypertension. Besides, clinicians are most concerned about renal artery stenosis ≥70%, because this part of patients may need further surgical treatment, and CEUS has an advantage in detecting these patients.

## Discussion

**Figure 3:**
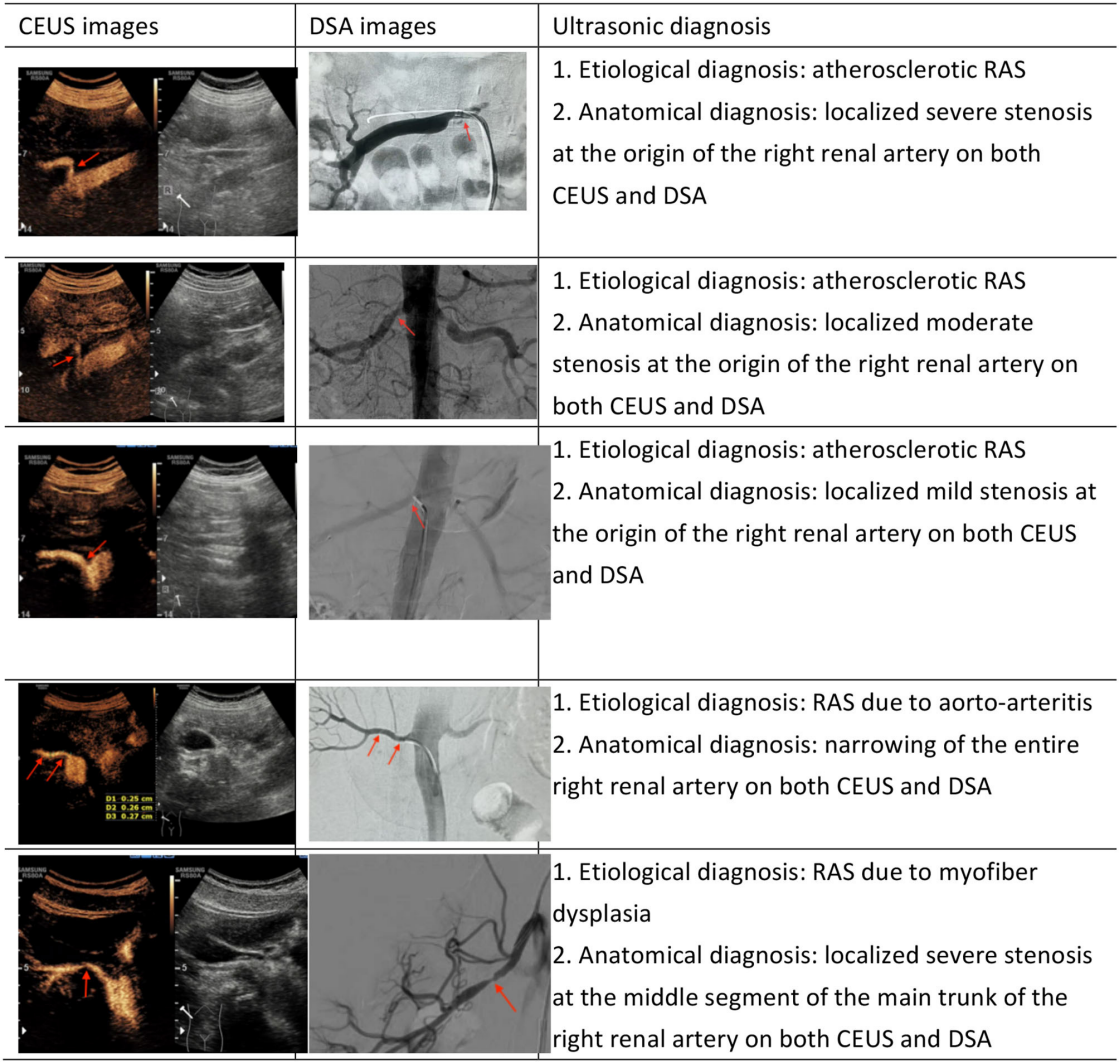
Comparison of RAS cases in CEUS and DSA (The red arrow in the figure shows the narrow position).

Currently, ultrasound assessments of RAS are mostly based on direct and indirect hemodynamic parameters derived by DUS, including PSV in the stenotic lumen, PSV in the abdominal aorta, RAR and the presence of tardus-parvus renal artery spectral waveforms. Assessments based on these parameters do not determine the degree of stenosis according to the changes in vascular luminal diameter. Morphological ultrasonic imaging can display the morphology of the renal artery lumen through a variety of ultrasound technologies. The morphological technologies commonly used in clinical practice include conventional grayscale imaging, two-dimensional bright-mode blood flow imaging (B-flow) (10), color Doppler flow imaging (CDFI) (11), power Doppler imaging (PDI) and CEUS. CDFI, a common technology in clinic practice, can locate a stenosis based on significant color aliasing at the stenotic site. However, this technology also has disadvantages, e.g., blood flow signal spillover and intraluminal blood flow signal loss related to the angle between the directions of the blood flow and the ultrasonic beam (e.g., when the angle is 90 degrees). PDI, which is not affected by the blood flow direction and the angle of incidence, can more sensitively display low-velocity blood flow and small blood vessels, but it cannot reflect blood flow properties and may have blood flow signal spillover (12, 13). CEUS can be used to visualize the main renal artery trunk by bolus injection of ultrasound contrast agents, like the processes and principles of DSA (Figure 3). However, as an invasive technology, DSA is not considered a routine screening method for RAS. In this study, we focused on the diagnostic accuracy rate of CEUS relative to DSA for RAS, as well as the reasons underlying missed diagnosis and misdiagnosis by CEUS. Among 80 renal arteries, one case of mild stenosis and one case of moderate stenosis were missed by CEUS. After reviewing the images, we found that the main reason for missed diagnosis by CEUS was related to the size and location of the plaques because the detection of small plaques is difficult with CEUS, especially plaques located in the lateral wall. In addition, renal arteries with mild or moderate stenosis often do not exhibit hemodynamic changes, limiting the diagnostic value of hemodynamic parameters for the diagnosis of RAS. Among 13 RAS misdiagnoses, three were caused by the blockage of acoustic shadows from the hyperechoic plaques in the vascular wall at the beginning of the renal artery. The blockage caused contrast agent echoes in the residual lumen to appear relatively narrow, leading to measurement inaccuracy (overestimation) by CEUS regarding the degree of stenosis. Two cases of misdiagnosis were related to vascular remodeling after aortic arteritis. In patients with remodeled renal arteries, the measured normal luminal diameter was small, leading to an overestimation of stenosis. When DUS parameters were taken into account, CEUS-based diagnoses of moderate stenosis were corrected to severe stenosis, consistent with the DSA results in four cases. There were mainly two scenarios in these four cases. One scenario was that the renal artery showed moderate stenosis on CEUS but demonstrated tardus-parvus waveforms on DUS, indicating severe stenosis; therefore, the final diagnosis was severe stenosis when all factors were considered. The other scenario was that the renal artery showed moderate stenosis on CEUS and no tardus-parvus waveforms on DUS but an RAR higher than three and an average velocity higher than 200 cm/s at all stenotic sites; therefore, the final diagnosis was severe stenosis when all factors were considered.

Ultrasound assessments for RAS are mainly based on morphological and hemodynamic parameters, and these two categories of parameters complement and verify each other; however, each has its own limitations. The presence of tardus-parvus renal artery spectral waveforms, which is a hemodynamic parameter, might lead to a false-positive diagnosis. For example, in patients with arteritis, the severely stenotic abdominal aorta could result in tardus-parvus waveforms in the intrarenal arteries at both sides, but the waveform pattern has limited diagnostic value and does not indicate severe stenosis of the main renal arteries at both sides. In addition, the hemodynamic changes in RAS, which involve a number of direct and indirect parameters, are relatively complex. These parameters are affected by both kidney lesions and systemic factors of patients. Therefore, applying these parameters for the diagnosis of RAS requires rational analysis and judgment. Instead, CEUS provides morphological information for diagnosis, more similar to the basic principles of DSA. Based on previous experience, ultrasound imaging of renal arteries is often difficult because the arteries travel in the retroperitoneal space and have a small diameter; therefore, the imaging quality heavily depends on the operator, requiring operators to have relatively well-developed skills. The patients at Beijing Hospital are mostly elderly, and most have accompanying diseases such as renal insufficiency; thus, CTA and MRA are not suitable. In the past four years, renal artery CEUS has been a research focus of our angiography group in the ultrasound department, with more than 700 patients undergoing CEUS. Compared with DSA, CEUS has a higher detection rate and diagnostic accuracy. Here, we briefly summarize some experience gained by our angiography team. Compared with conventional ultrasound, CEUS is more sensitive for displaying blood flow and can clearly show the main renal artery trunk by using contrast agents. The contrast agent echoes of a normal renal artery trunk exhibit a natural traveling course with a generally consistent width and intensity. Special attention should be paid to the following three aspects when performing CEUS of the renal arteries. The first aspect is the body position of the patient. The patient should be scanned for the observation and measurement of the renal arteries in all three positions, namely, the supine, right lateral, and left lateral decubitus positions. Based on our experience, the coronal plane on the lateral side of the waist is the first choice for CEUS scanning, for which a specially designed cushion can be used to stabilize the patient's position. The patient's back should form a 60 to 90 degree angle with the operating bed when he/she is in the right lateral decubitus position and a 45 to 60 degree angle when he/she is in the left lateral decubitus position. The second aspect is the scanning section. After the patient is fixed in the lateral decubitus position and the long axis of the abdominal aorta is displayed as much as possible, the ultrasound probe should be pressed deeply toward the direction of the main renal artery to allow clear illustration of the beginning of the renal artery. To avoid the blockage of contrast agent echoes by plaques at the base of the renal artery and allow the determination of whether an accessory renal artery originates from the abdominal aorta either above or below the origin of the renal artery, the probe should be positioned to ensure, as much as possible, that the traveling course of the proximal renal artery is parallel to the acoustic beam. The third aspect is the timing of contrast-enhanced scanning. The main renal artery trunk should be observed during the arterial phase of angiography while avoiding accompanying renal veins as much as possible.

The 40 patients in this study were elderly with an average age of approximately 60 years old, and were overweight with an average BMI of about 24.20 kg/m2. However, the sonographers, who were professionally trained for standardized operation, were able to clearly display renal arteries on CEUS especially in the detection of renal artery stenosis, and the detection of renal artery stenosis which is ≥70%, CEUS and DSA have good consistency. All this suggests the necessity and importance of standardized operation of renal artery contrast-enhanced ultrasound examination.

In conclusion, it is critical for diagnostic medical sonographers to possess abilities that providing not only anatomical diagnoses of renal arteries but also etiologic diagnosis by incorporating information such as patient age, sex, medical history, and various laboratory findings. In addition, CEUS can semi-quantitatively determine the status of cortical renal perfusion and reflect renal function by using the parameters of peak intensity, time to peak, slope of the ascending branch, mean transit time, and area under the curve based on time-intensity curves. This will be a research direction of our group in the future.

## Data Availability Statement

The original contributions presented in the study are included in the article/supplementary material, further inquiries can be directed to the corresponding author/s.

## Ethics Statement

The studies involving human participants were reviewed and approved by Institutional Review Board of Beijing Hospital. The patients/participants provided their written informed consent to participate in this study.

## Author Contributions

YW wrote the manuscript. JR edited the manuscript. All authors contributed to the article and approved the submitted version.

## Funding

This study was supported by grants from the Beijing Hospital Clinical Research 121 Project (No. BJ-2018-198) and the Scientific Research Project of Beijing Hospital (No. 2018-001).

## Conflict of Interest

The authors declare that the research was conducted in the absence of any commercial or financial relationships that could be construed as a potential conflict of interest.

## Publisher's Note

All claims expressed in this article are solely those of the authors and do not necessarily represent those of their affiliated organizations, or those of the publisher, the editors and the reviewers. Any product that may be evaluated in this article, or claim that may be made by its manufacturer, is not guaranteed or endorsed by the publisher.
